# Chiral MoS_2_@BC fibrous membranes selectively promote peripheral nerve regeneration

**DOI:** 10.1186/s12951-024-02493-6

**Published:** 2024-06-17

**Authors:** Mengru Li, Xiao Li, Yaowei Lv, Hede Yan, Xiang-Yang Wang, Jin He, Chao Zhou, Yuanming Ouyang

**Affiliations:** 1 College of Fisheries and Life Science, Shanghai Ocean University, Shanghai, 201306 China; 2 Department of Orthopedics, Shanghai Sixth People’s Hospital Affiliated to Shanghai Jiao Tong University School of Medicine, Shanghai, 200233 China; 3 Shanghai Engineering Research Center for Orthopaedic Material Innovation and Tissue Regeneration, Shanghai, 201306 China; 4 Department of Orthopaedics, The Second Affiliated Hospital of Wenzhou Medical University, Wenzhou, 325000 China; 5 Department of Pediatric Orthopaedics, Xinhua Hospital, School of Medicine, Shanghai Jiao Tong University, Shanghai, 200092 China

**Keywords:** Chiral nanomaterials, Molybdenum disulfide, Peripheral nerve injury, Biocompatibility, Effectiveness

## Abstract

**Background:**

Molybdenum disulfide (MoS_2_) has excellent physical and chemical properties. Further, chiral MoS_2_ (CMS) exhibits excellent chiroptical and enantioselective effects, and the enantioselective properties of CMS have been studied for the treatment of neurodegenerative diseases. Intriguingly, left- and right-handed materials have different effects on promoting the differentiation of neural stem cells into neurons. However, the effect of the enantioselectivity of chiral materials on peripheral nerve regeneration remains unclear.

**Methods:**

In this study, CMS@bacterial cellulose (BC) scaffolds were fabricated using a hydrothermal approach. The CMS@BC films synthesized with L-2-amino-3-phenyl-1-propanol was defined as L-CMS. The CMS@BC films synthesized with D-2-amino-3-phenyl-1-propanol was defined as D-CMS. The biocompatibility of CMS@BC scaffolds and their effect on Schwann cells (SCs) were validated by cellular experiments. In addition, these scaffolds were implanted in rat sciatic nerve defect sites for three months.

**Results:**

These chiral scaffolds displayed high hydrophilicity, good mechanical properties, and low cytotoxicity. Further, we found that the L-CMS scaffolds were superior to the D-CMS scaffolds in promoting SCs proliferation. After three months, the scaffolds showed good biocompatibility in vivo, and the nerve conducting velocities of the L-CMS and D-CMS scaffolds were 51.2 m/s and 26.8 m/s, respectively. The L-CMS scaffolds showed a better regenerative effect than the D-CMS scaffolds. Similarly, the sciatic nerve function index and effects on the motor and electrophysiological functions were higher for the L-CMS scaffolds than the D-CMS scaffolds. Finally, the axon diameter and myelin sheath thickness of the regenerated nerves were improved in the L-CMS group.

**Conclusion:**

We found that the CMS@BC can promote peripheral nerve regeneration, and in general, the L-CMS group exhibited superior repair performance. Overall, the findings of this study reveal that CMS@BC can be used as a chiral nanomaterial nerve scaffold for peripheral nerve repair.

**Graphical Abstract:**

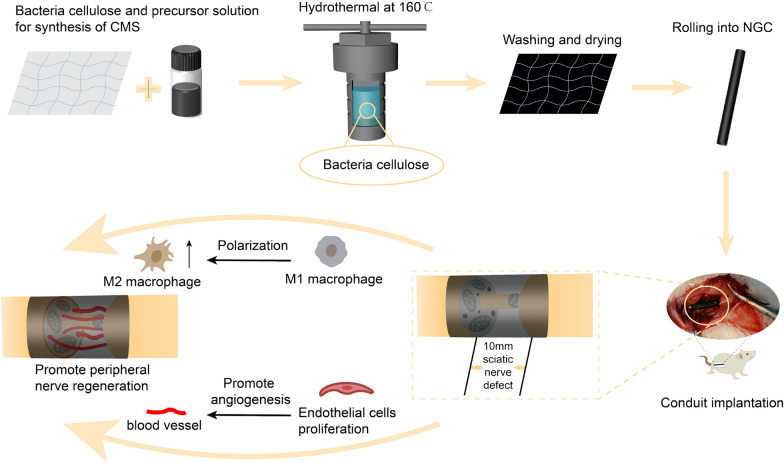

**Supplementary Information:**

The online version contains supplementary material available at 10.1186/s12951-024-02493-6.

## Introduction

Approximately one million people worldwide suffer from peripheral nerve injury each year [1]. These nerve injuries typically lead to motor dysfunction and place a heavy economic burden on the healthcare system [2]. When a damaged nerve gap in the peripheral nervous system is less than 5 mm, it can naturally regenerate [3]. However, when there is a large gap between the nerve ends, direct suture of the nerve gap usually causes tension on the two nerve ends [4]. When the nerve defect is longer than 10 mm, autologous nerve transplantation or the involvement of tissue engineering techniques are necessary [5, 6]. Autologous nerve transplantation is the gold standard when the nerve defect is more than 2–3 cm [7], but there are several shortcomings, such as the lack of donor nerve, donor site damage and the risk of neuroma formation [5, 8]. Therefore, tissue-engineering technologies have been developed to provide new routes for peripheral nerve regeneration.

Nerve guidance conduits (NGCs) provide physical support that promotes nerve regeneration [9]. Natural polymers, including chitosan and collagen, have been used in NGCs because of their good biocompatibility and biodegradability [10–12]. Chitosan NGCs are already in use in Europe and USA as scaffolds for peripheral nerve regeneration. However, some natural polymers have weak mechanical properties [13]. Composite polymer scaffolds possess both good mechanical properties and cellular affinity, and they have been extensively studied for neural tissue engineering. Different nerve conduits can promote the repair of injured peripheral nerves by different mechanisms. Neural tissue regeneration requires blood vessels to ensure sufficient oxygen and nutrient. Qian et al. demonstrated that black phosphorus nano-scaffold induced angiogenesis and neurogenesis [14]. Mitsuzawa et al. showed that bio three-dimensional (3D) conduit composed of xenofree human induced pluripotent stem cell–derived mesenchymal stem cells (iMSCs) can promote angiogenesis [15]. Studies have showed that topographical cues can promote peripheral nerve regeneration [7, 16–18]. Moreover, the excessive inflammatory response can be harmful to the repair of damaged nerves. Regulating immune balance facilitates the repair of damaged nerves. Wang et al. showed that the dimethyl fumarate/regenerated silk fibroin/poly (3,4-ethylenedioxythiophene): poly(styrene sulfonate) conduits can reduce inflammatory factors’ release and have good ability to promote nerve regeneration [19]. Qian et al. showed that the melatonin/polycaprolactone nerve guide conduit can inhibit oxidative stress and inflammation after traumatic insults [9]. Yang et al. demonstrated that bionic peptide hydrogel scaffolds can promote M2 transformation in situ and lead to proliferation and migration of Schwann cells, neuron growth and motor function recovery [8]. Appropriate electrical stimulation can be beneficial in promoting nerve regeneration. Zhang et al. showed that poly(ɛ-caprolactone)/carbon nanotubes composite fiber can promote neural regeneration by electrical stimulation [20]. Zhao et al. showed that polypyrrole/silk fibroin conductive composite scaffolds can promote axonal regeneration and remyelination in vivo by electrical stimulation [21]. Park et al. showed that r (graphene oxide/gelatin methacryloyl) NGC can promote peripheral nerve regeneration by delivering electrical signals [22].

Molybdenum disulfide (MoS_2_) is recognized as a kind of promising material for tissue engineering, and several studies have shown that MoS_2_ can promote the repair of cardiac, neural, and skin tissues [23–25]. MoS_2_-thread-based scaffold possesses good biocompatibility in vitro [26]. In particular, nanostructured MoS_2_ thin films facilitate the attachment of neural stem cells (NSCs) and promote their differentiation into neurons [27]. Moreover, MoS_2_-thread-based scaffold can be used as an attractive material for nerve tissue regeneration due to its good conductivity and good biocompatibility [26]. Chen et al. showed that MoS_2_/graphite oxide/polyvinyl alcohol composite hydrogel can promote nerve cells growth and promote the differentiation of neural stem cells into neural cells [28]. The ultrathin MoS_2_−MoO_3−*x*_ heterojunction nanosheets can promote the differentiation of human neural progenitor cells (hNPCs) into nervous lineages [29]. Furthermore, sulfur and molybdenum are essential elements for organisms [30, 31]. On the basis of these earlier studies, in this study, we integrated MoS_2_ nanomaterials with chiral materials to develop a new type of compound scaffold for nerve regeneration.

Chiral nanomaterials are well-known for their excellent chiroptical and chirality-dependent characteristics [32]. These properties have facilitated their widespread use in tissue engineering [33–35]. For example, Shi et al. showed that chiral nanoparticles had a therapeutic effect on Alzheimer’s disease by facilitating the differentiation of mouse NSCs into neurons [36]. Zhang et al. found that l-cysteine-anchored gold nanoparticles (AuNPs) had a better therapeutic effect on promoting periodontal regeneration than d-cysteine-anchored AuNPs [37]. Xu et al. reported that chiral AuNPs could alleviate Parkinson’s disease by eliminating senescent microglial cells [38]. Furthermore, etanercept-MoS_2_@poly (ethylene glycol) nanoflowers can treat spinal cord injury (SCI) by promoting macrophage M2 polarization [39]. However, the role of chiral MoS_2_ (CMS) nanomaterials in peripheral nerve regeneration has not yet been studied.

**Fig. 1:**
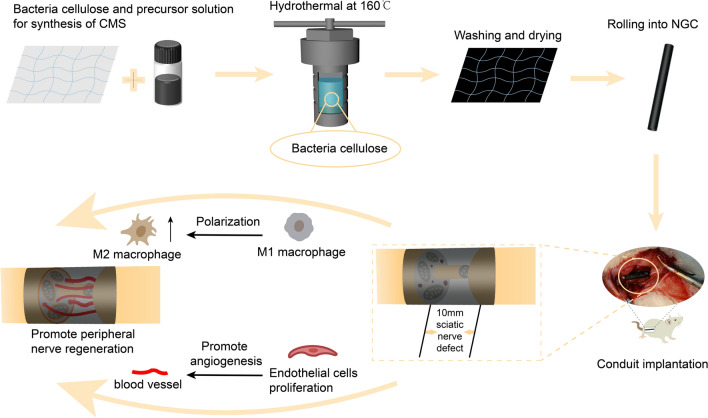
NGC production and implantation in a 10-mm rat sciatic nerve defect model

In this study, CMS@bacteria cellulose (BC) scaffolds were synthesized using a hydrothermal approach (Fig. 1). The CMS@BC scaffolds were characterized and used for peripheral nerve regeneration. In addition, we evaluated their biosafety by culturing Schwann cells (SCs) on the scaffolds and implanting the scaffolds into rats for three months. Finally, we evaluated their ability to repair the sciatic nerve and their potential to restore local motor and electrophysiological activity rapidly.

## Materials and methods

### Preparation of five types of membranes

Thioacetamide (CH_3_CSNH_2_; 99.0%) and sodium molybdate dihydrate (Na_2_MO_3_⋅2H_2_O, 99.0%) were supplied by Merck Reagent Co., Ltd (China). D-, L- and racemic-2-amino-3-phenyl-1-propanol (APP, 99.9%) were obtained from Tansoole Co., Ltd. (Shanghai, China). The BC fibrous membranes were obtained from Guilin Qihong Technology Co., Ltd. (China). All reagents were used without purification. All water used was ultrapure (18.2 MΩ/cm) and prepared using a Heal Force SMART Ultra-pure water system.

#### Substrate treatment

The BC fibrous membranes were soaked in a large amount of deionized water and then washed with ethanol for three times in an ultrasonic environment.

#### Preparation of CMS@BC

A previously reported method for the synthesis of chiral inorganic materials was used to prepare the CMS@BC with a well-controlled chiral structure [40–44]. In a typical procedure, sodium molybdate dihydrate (2 mmol) and thioacetamide (1 mmol) were dissolved in H_2_O (25 mL), denoted A. Then, APP (2 mmol) was dissolved in H_2_O (5 mL), denoted B. Next, solution B was quickly added to solution A with vigorous magnetic stirring. The BC fibrous membranes were then added to the mixed solution, and the mixture was stirred for 30 mins. The magnetic field was removed, and the solution was transferred to an autoclave. The container was then closed and maintained at 180 °C for 6 h. The autoclave was allowed to cool naturally to room temperature. The sections were washed several times with deionized water and ethyl alcohol and freeze-dried overnight.

**Fig. 2:**
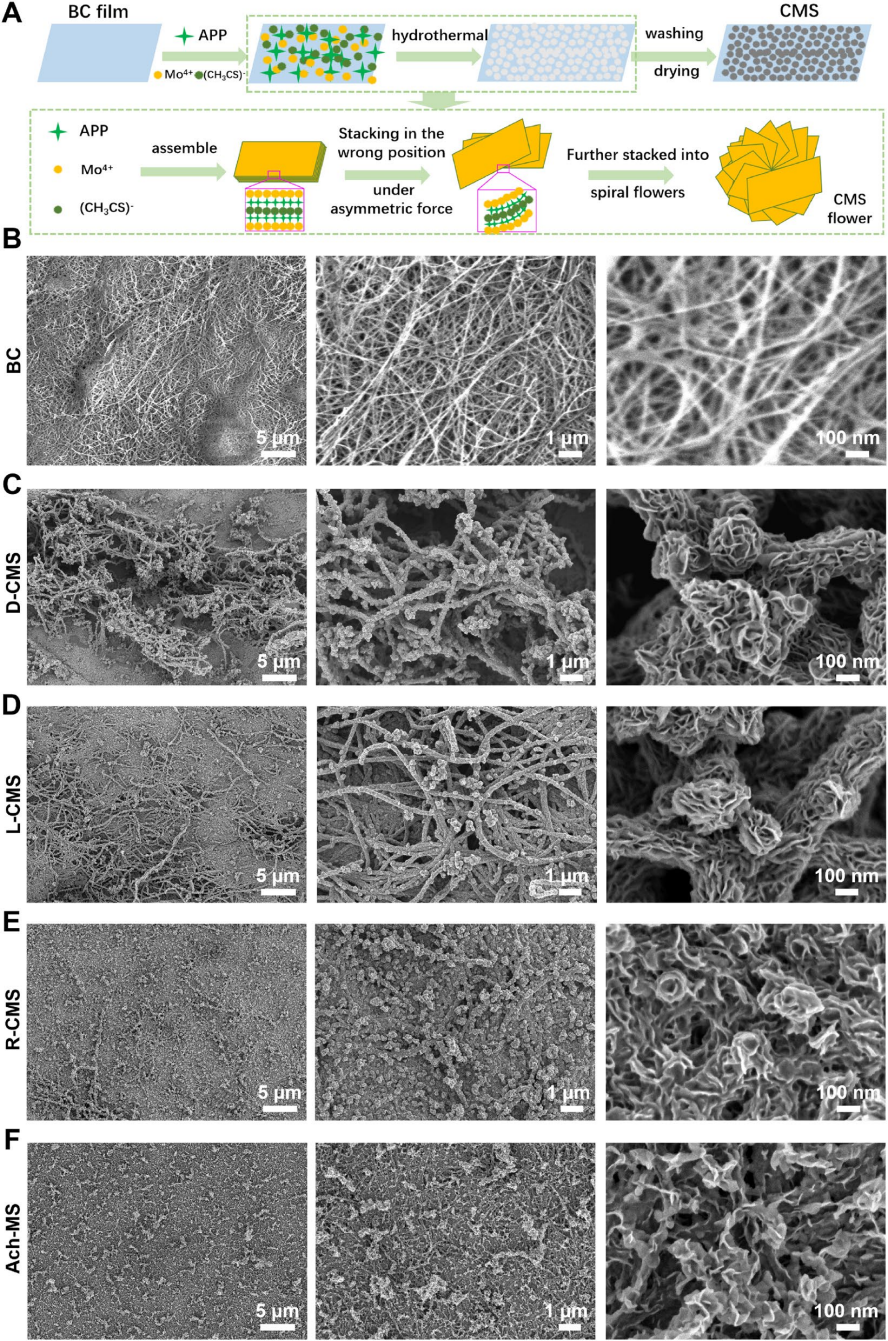
Morphology and chiral structure of BC, D-CMS, L-CMS, Ach-MS and R-CMS films. **A** Schematic of the synthesis process for CMS@BC films. **B–F** SEM images of BC **B,** D-CMS **C,** L-CMS **D,** R-CMS **E**, and Ach-MS **F** at varying magnifications. The synthetic molar composition was 1 APP:1 Na_2_MO_3_⋅2H_2_O:0.84 CH_3_CSNH_2_:1800 H_2_O

CMS nanoplates were deposited on a BC fibrous membrane using a facile chiral-APP-induced self-assembly strategy (Fig. 2A). The fibrous BC membranes were cleaned with compressed air, and CMS nanoplates were deposited on the membranes from a homogeneous solution composed of APP, sodium molybdate dihydrate, thioacetamide, and deionized water under hydrothermal conditions. L-, D-, and Rac-APP were chosen as the structure-directing and symmetry-breaking agents for the asymmetric attachment and co-self-assembly process with Mo^6+^ ions because of their complexation behavior. Crucially, ammonium persulfate decomposes slowly, thus controlling the release of sulfur ions. Hence, the Mo^6+^ in the solution precipitated slowly on the breakdown of ammonium persulfate, forming an alkaline environment and subsequently yielding chiral MoS_2_ nanosheets. The CMS@BC films were collected by removing the BC fibrous membranes from the synthetic solution. The CMS@BC films synthesized using L-APP, D-APP, and Rac-APP were denoted L-CMS, D-CMS, and R-CMS, respectively. The CMS@BC films synthesized with free chiral molecules were denoted Ach-MS. The CMS@BC films were used as nerve defect repair materials.

### Characterization

#### X-ray powder diffractometry (XRD)

XRD patterns were recorded using a Bruker X-ray diffractometer D2 Phaser with Cu-Kα radiation (40 kV, 30 mA, λ = 0.15418 nm) at a rate of 5°/min over the range of 10°-80°.

#### Scanning electron microscopy (SEM)

The morphologies of the samples were observed using a JEOL JSM-7900F scanning electron microscope with an accelerating voltage of 1.0 kV and a working distance of 4 mm to minimize charge accumulation.

#### Circular dichroism spectrometer (CD)

DRCD and UV/Vis spectra were obtained using a JASCO J-1500 spectropolarimeter fitted with a DRCD apparatus. The scaffolds were positioned between the normally incident light and a black backboard. All the reflected light was collected by an integrating sphere before reaching the CD detector.

#### Contact angle (CA) measurements

The material hydrophilicity was determined using a SINDIN SDC-350H contact angle meter. The solid or film sample was fixed to the test bench so that its surface was parallel to that of the test bench. The appropriate liquid was selected and added to the syringe, and a droplet was extruded by fine-tuning the valve so that it was suspended on the surface of the sample without falling. The camera was turned toward the droplet, and a picture of the contact line of the droplet with the surface was taken.

#### Mechanical properties

The material mechanical properties were determined by a tensile testing machine (Tophung, TH-8100ST, China).

### SCs viability and proliferation assay

SCs were purchased from Zhongqiaoxinzhou Biotech (Shanghai, China) and cultured in Dulbecco’s Modified Eagle’s Medium (Gibco, USA) supplemented with 1% penicillin/streptomycin solution (Gibco, USA) and 10% fetal bovine serum (Gibco, USA) in an incubator (37 ℃, 5% CO_2_). All membranes were soaked in alcohol and exposed to ultraviolet light for 12 h for sterilization. Next, 1 × 10^5^ SCs were cultured on the BC, D-CMS, L-CMS, Ach-MS, and R-CMS membranes. One day later, the viability of the SCs was assessed using a live/dead (Solarbio, China) cell assay. Furthermore, we used Annexin V-FITC (Absin, Shanghai, China) apoptosis assays to evaluate cell viability after SCs have grown on the five kinds of materials for two days. Fluorescence microscope (Leica, Germany) was used to collect the results of the live/dead cell assay. The experimental results were analyzed using ImageJ (USA). We used FlowJo (USA) to analyze the results of the Annexin V-FITC assays. The effects on cell proliferation were examined using the Cell Counting Kit 8 (CCK8) (Absin, China) proliferation assay.

### Immunofluorescence assay

SCs were cultured on the five types of scaffolds for four days. Subsequently, the SCs were washed with phosphate buffer saline (PBS; Servicebio, China). The SCs were then fixed with 4% paraformaldehyde (PFA; Biosharp, China), soaked in Triton X-100 (Solarbio, China), and blocked with bovine serum albumin (Solarbio, China). Primary and secondary antibodies were sequentially incubated with SCs. The nuclei were then stained with DAPI (Beyotime, China). The primary antibodies used were anti-S100β (1:250; Abcam, USA) and anti-Ki67 (1:250; Abcam, USA). Images were acquired using a fluorescence microscope (Leica, Germany).

### Animal surgery

Thirty male Sprague–Dawley (SD) rats (250–300 g) were randomly divided into six groups (n = 5). The rats were anesthetized by intraperitoneal injection of an appropriate amount of pentobarbital sodium. Then, a 10-mm segment of the sciatic nerve was removed from the right hind limb, and a 12-mm-long NGC was sutured to both ends of the cut nerve. In the autograft group, the transected sciatic nerve was rotated 180° and sutured to both ends of the severed nerve. The SD rats were then placed back in their cages and monitored. The animal experiments were conducted in accordance with the criteria established by the Animal Ethics Committee of Shanghai Shengchang Biotechnology Co., Ltd. (No. 2022–11-DLRMYY-OYYM-039).

### Functional recovery assay

Twelve weeks post-implantation, functional recovery tests were carried out. Rats were allowed to walk on a walk-trace analysis machine (Shanghai, China). The length from the second toe to the fourth toe (IT), the first toe to the fifth toe (TS), and the heel to the third toe (PL) were automatically recorded by a computer and analyzed using VisuGait. The sciatic functional index (SFI) was calculated using the following formula: SFI = (− 38.3 × (EPL-NPL) ÷ NPL) + (109.5 × (ETS-NTS) ÷ NTS) + (13.3 × (EIT-NIT) ÷ NIT)  − 8.8. Here, 'E' denotes the experimental feet and 'N' denotes the normal feet. The SFI values range from -100 to 0. A value of 0 represents full function, whereas a value of -100 represents loss of function.

### Electrophysiology assessment

The electrophysiological properties were characterized 12-weeks after implantation. Pentobarbital sodium was used to anesthetize rats. The sciatic nerves of the rats were exposed, and a recording electrode was inserted into the gastrocnemius muscle for electromyography. The nerve conducting velocity (NCV) was recorded by placing electrodes at the ends of the nerve.

### Morphological recovery assay

After the electrophysiological results were measured, the animals were euthanized with an overdose of anesthetic. All regenerated sciatic nerves were cut and fixed in 2.5% glutaraldehyde solution and 4% PFA, respectively. Transmission electron microscopy (TEM, Thermo Fisher Talos L120C) was used to observe the resected nerve samples, and others were stained with toluidine blue (TB). The main organs (heart, liver, spleen, lung, and kidney) were fixed with 4% PFA and stained with hematoxylin and eosin (HE). The gastrocnemius muscles were sliced into sections and stained with HE. ImageJ was used to calculate the average area of the muscle fibers.

### Statistical analysis

The experimental results are presented as mean ± standard deviation. A student’s t-test and one-way analysis of variance (ANOVA) were used for statistical analysis by GraphPad Prism 9. And then we used the “multiple comparisons” in the ANOVA analysis to assess differences between the groups. Differences between L-CMS and other groups were considered significant at *p < 0.05, **p < 0.01, ***p < 0.001, and ****p < 0.0001.

## Results

### Fabrication and characterization of scaffolds

#### Synthesis of CMS@BC

In this study, D-CMS, L-CMS, Ach-MS, and R-CMS films were fabricated using a hydrothermal approach. The antipodal CMS@BC films were black and had a fiber-network structure. Figures 2B–F show the SEM images of the BC, D-CMS, L-CMS, R-CMS, and Ach-MS films. BC had a porous network structure comprising nanofibers (Fig. 2B), whereas CMS had a flower-like structure with assembled nanosheets distributed on the surface of the BC fibrous membranes. On the L-CMS@BC films, the L-CMS flowers were composed of densely arranged nanoplates having a width of 50–100 nm, thickness of 5–10 nm, and height of 20–100 nm, which grew on the surface of the BC fibers. Left-handed helical stacking of the nanoplates (Fig. 2D) was observed, which indicated the third level of chirality. The magnified SEM image (Fig. 2D) reveals a wave-twisting pattern in the nanoplates, which represents the secondary level chirality of CMS nanoplate. Based on our previous study [45], we believe that the misalignment of atoms is responsible for the first level of chirality. Similarly, right-handed helical stacking of nanoplates was observed in the D-CMS flowers in the D-CMS@BC films (Fig. 2C), and this is the tertiary level of chirality. The magnified SEM image also shows the twisting, wavelike pattern of the nanoplates. The presence of a right-handed twisted structure in the nanoplates is considered to be the secondary level of chirality of the D-CMS flowers (Fig. 2C). The first level of chirality was also right-handed [45]. The R-CMS flowers on the R-CMS@BC films (Fig. 2E) revealed randomly arranged nanoplates having irregular structures. In contrast, the Ach-MS (Fig. 2F) membrane presented a disorganized stacked nanosheet structure, which was not assembled into ordered flowers, and therefore did not exhibit a chiral multilevel structure.

**Fig. 3:**
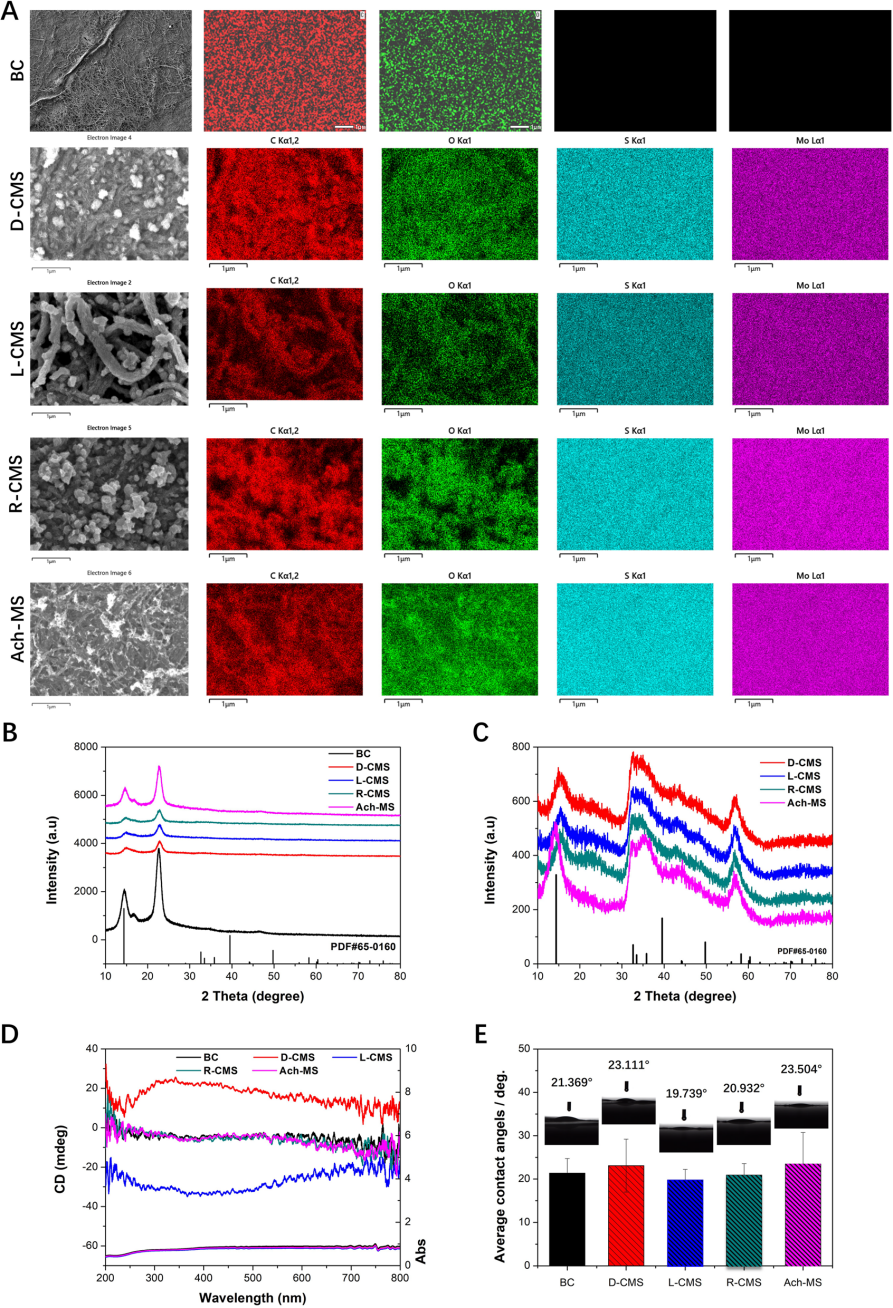
Element distribution, multiple OAs, and crystal structure of the BC, D-CMS, L-CMS, Ach-MS and R-CMS films. **A** EDS mapping of BC, D-CMS, L-CMS, R-CMS, and Ach-MS. Scale bar: 1 µm. **B, C** XRD patterns of CMS@BC films and CMS powder. Characteristic MoS_2_ reflections are labelled with the standard card reflections (λ = 1.5418 Å; JCPDS No. 65–0160). **D** DRUV-Vis and DRCD spectra of the chiral CMS@BC films shown in Fig. 3 measured with a black background. **E** CAs of different CMS@BC films

Figure 3A showed the energy-dispersive spectroscopy (EDS) maps of the surface of the BC substrate and CMS@BC film specimens, revealing the homogeneous presence of Mo, S, O, and C. The wide-angle X-ray diffractometry (XRD) pattens of the CMS@BC films and BC substrate were shown in Fig. 3B. Although the BC substrate yielded more intense peaks in the XRD pattern of CMS@BC, the characteristic reflections of the hexagonal phase of CMS (space group P63/mmc; a = b = 3.161 Å and c = 12.295 Å [JCPDS file 65–0160]) can still be identified. The XRD pattern of CMS powder was also examined. As shown in Fig. 3C, the characteristic peaks of CMS match those in the standard PDF card. The chirality of the CMS@BC films was unequivocally determined by employing diffuse reflectance ultraviolet-visible (DRUV-Vis) and diffuse reflectance circular dichroism (DRCD) spectroscopy, as shown in Fig. 3D. These methods were chosen because of the opaqueness of the scaffolds, thus ensuring the accurate detection of optical activity (OA). The DRCD with white and black backgrounds predominantly exhibits absorption-based optical activity (AOA), whereas both scattering-based optical activity (SOA) and AOA were observed because of the reflection of almost all visible light by the white background and absorption by the black backboard, respectively. Notably, the nanounits of the semiconductor aggregate in a chiral manner, exhibiting distances shorter than the Bohr exciton radius. This arrangement induces a dissymmetric field on the entire aggregate through excitation delocalization, thereby resulting in AOA based on electronic transitions [46]. The SOA occurred at multiple integral wavelengths based on mλ = Pn_avg_, where m is an integer, n_avg_ is the average refractive index, and P is the pitch length of the chiral medium [47]. A left-handed structure exhibits a preference for absorbing right-handed circularly polarized light and reflecting left-handed light, leading to the generation of negative AOA and SOA  signals during the DRCD measurement. As Fig. 3D showed, the L-CMS and D-CMS films exhibited symmetrical signals within the wavelength range of 200-800 nm. Taking L-CMS as an example, a broad band was observed in the UV–Vis spectrum, accompanied by a prominent peak in the CD spectrum within the same wavelength range. The OA of CMS@BC films included significant SOA because the black carbon substrate was perceived as a white background. The mirror-image DRCD spectrum of the CMS@BC films revealed inverted chirality. The DRCD spectra of the BC substrate confirmed that the observed OA of the CMS@BC films originated from hierarchical chiral structures rather than the BC substrates. The R-CMS exhibited a morphology similar to that of an antipodal CMS flower, albeit lacking chirality. The CD spectrum of Ach-MS also indicates that it lacks a chiral structure. Figure 3E illustrates the exceptional hydrophilicity of BC and CMS@BC films, suggesting their potential for biological applications. The results of mechanical property tests showed that the tensile strength of the pure BC film was 4.17 MPa, which was much smaller than that of several chiral films with MoS_2_ attached to the surface. The results showed that the MoS_2_ attached to the surface can greatly enhance the tensile properties of the film, which were basically higher than 12 MPa, and can be increased 3–6 times on the basis of pure BC film. This fully meets the needs of nerve repair materials. (Additional file 1: Fig. S1).

### Biocompatibility

**Fig. 4:**
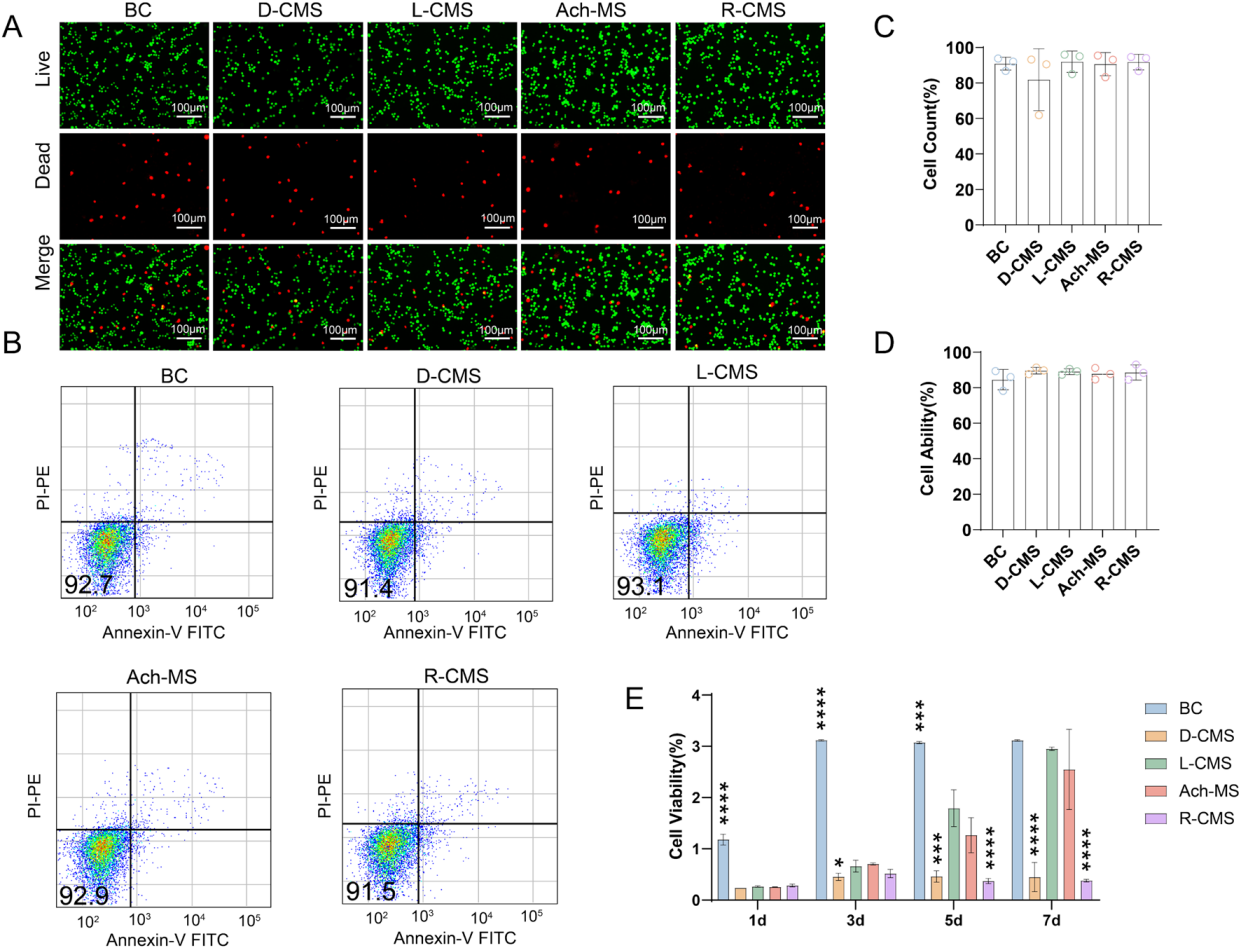
Cytocompatibility evaluation on BC, D-CMS, L-CMS, Ach-MS and R-CMS scaffolds. **A** Live/dead cell staining of SCs cultured on the scaffolds for 1 day. **B** Annexin V-FITC apoptosis experiments of SCs cultured on the scaffolds for 2 days. **C** Statistics for live/dead cell staining experiments. **D** Statistics for Annexin V-FITC apoptosis experiments. **E** CCK8 SC proliferation assay (*n* = 3)

SCs (1 × 10^5^) were cultured on the five types of scaffolds (BC, D-CMS, L-CMS, Ach-MS, and R-CMS), and live/dead experiments to test cell viability were carried out. As shown in Fig. 4A, the staining results for the five scaffolds indicated a high proportion of living cells and low proportion of dead cells (Fig. 4C). The results indicated that CMS@BC scaffolds did not exert prominent toxicity to the cells. We used the Annexin V-FITC assay to explore the biocompatibility of the different scaffolds (Fig. 4B) and found no significant differences in the rates of apoptosis of the five scaffolds (p > 0.05) (Fig. 4D), indicating similar cell survival rates. In addition, the cell proliferation on the different scaffolds was evaluated using the CCK8 assay, revealing that the L-CMS scaffolds promoted SCs proliferation better than D-CMS scaffolds when SCs were cultured on different materials for a long period of time (Fig. 4E).

### Immunofluorescence

**Fig. 5:**
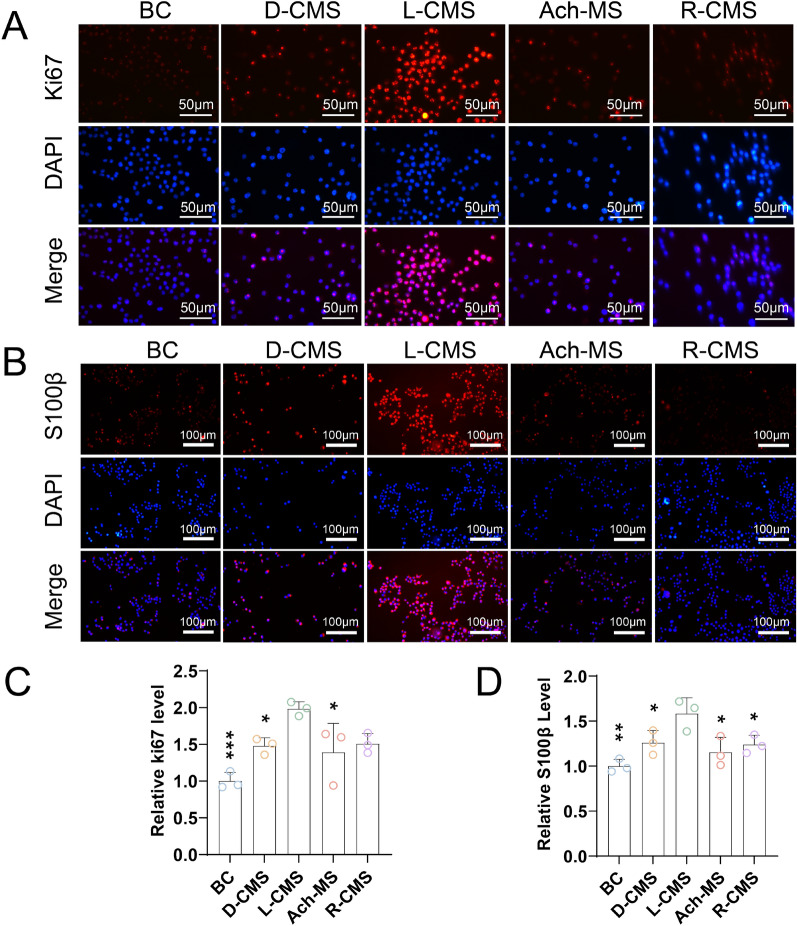
Immunofluorescence of SCs on BC, D-CMS, L-CMS, Ach-MS and R-CMS scaffolds. **A** Ki67 immunofluorescence and **B** S100β immunofluorescence. **C ** Relative levels of Ki67. **D** Relative levels of S100β (*n* = 3)

The Ki67 immunofluorescence results indicated that the L-CMS scaffolds promoted cell proliferation more effectively than the D-CMS scaffolds (p = 0.0403) (Fig. 5A and C). Here, S100β is the SC marker. The S100β immunofluorescence of the D-CMS scaffolds were significantly lower than that of the L-CMS scaffolds (p = 0.0484) (Fig. 5B and D).

### Biocompatibility in vivo

**Fig. 6:**
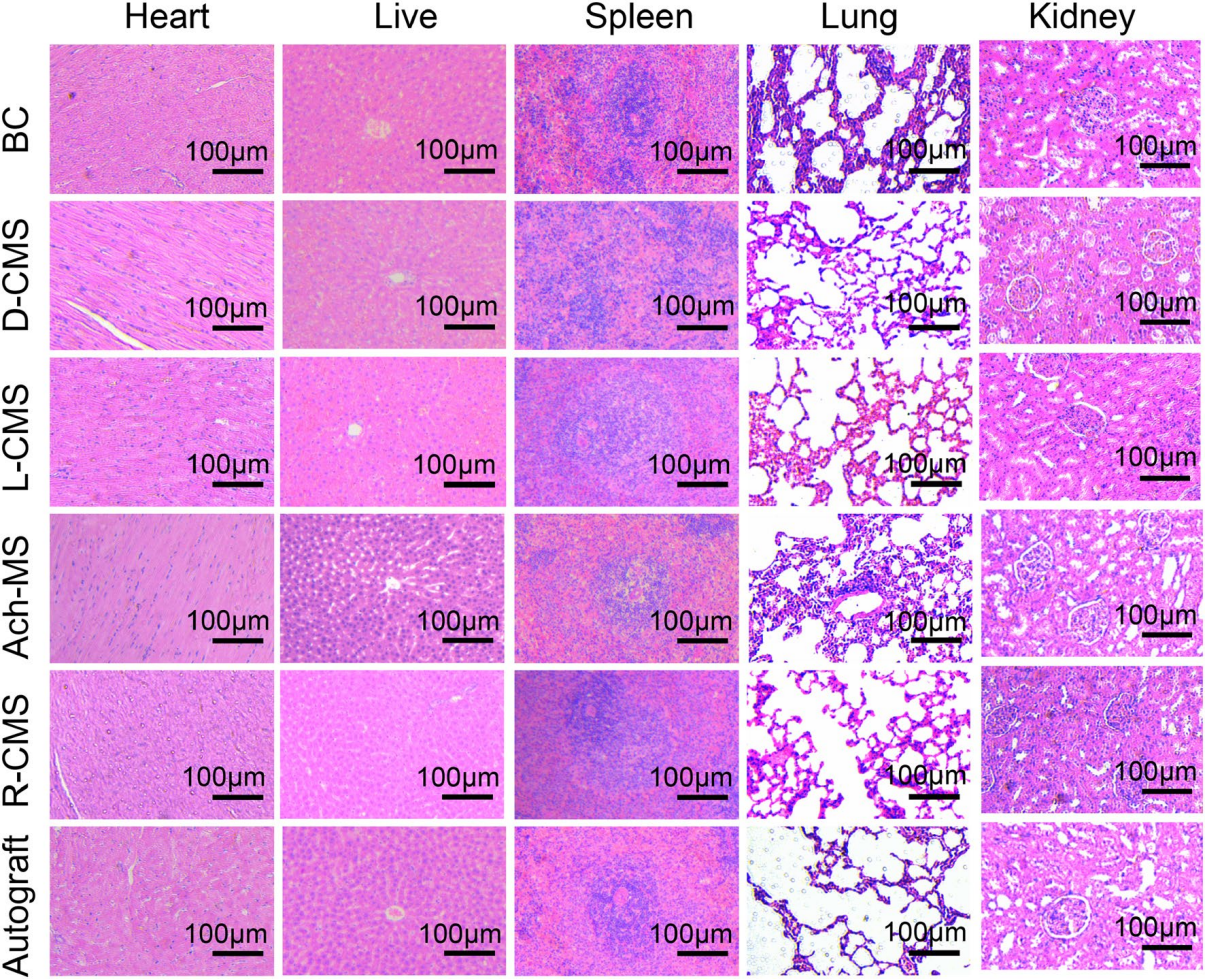
Toxicity evaluation in vivo. HE staining of main organs of rats in BC, D-CMS, L-CMS, Ach-MS, R-CMS and autograft groups as shown (*n* = 3)

The scaffolds were sutured to both ends of damaged sciatic nerves for 12 weeks. After this period, none of the rats had suffered infections or died. The results of HE staining of the rat main organs revealed no noticeable toxicity, suggesting that these scaffolds have excellent histocompatibility in vivo (Fig. 6).

### Chiral NGCs promote nerve function restoration

**Fig. 7:**
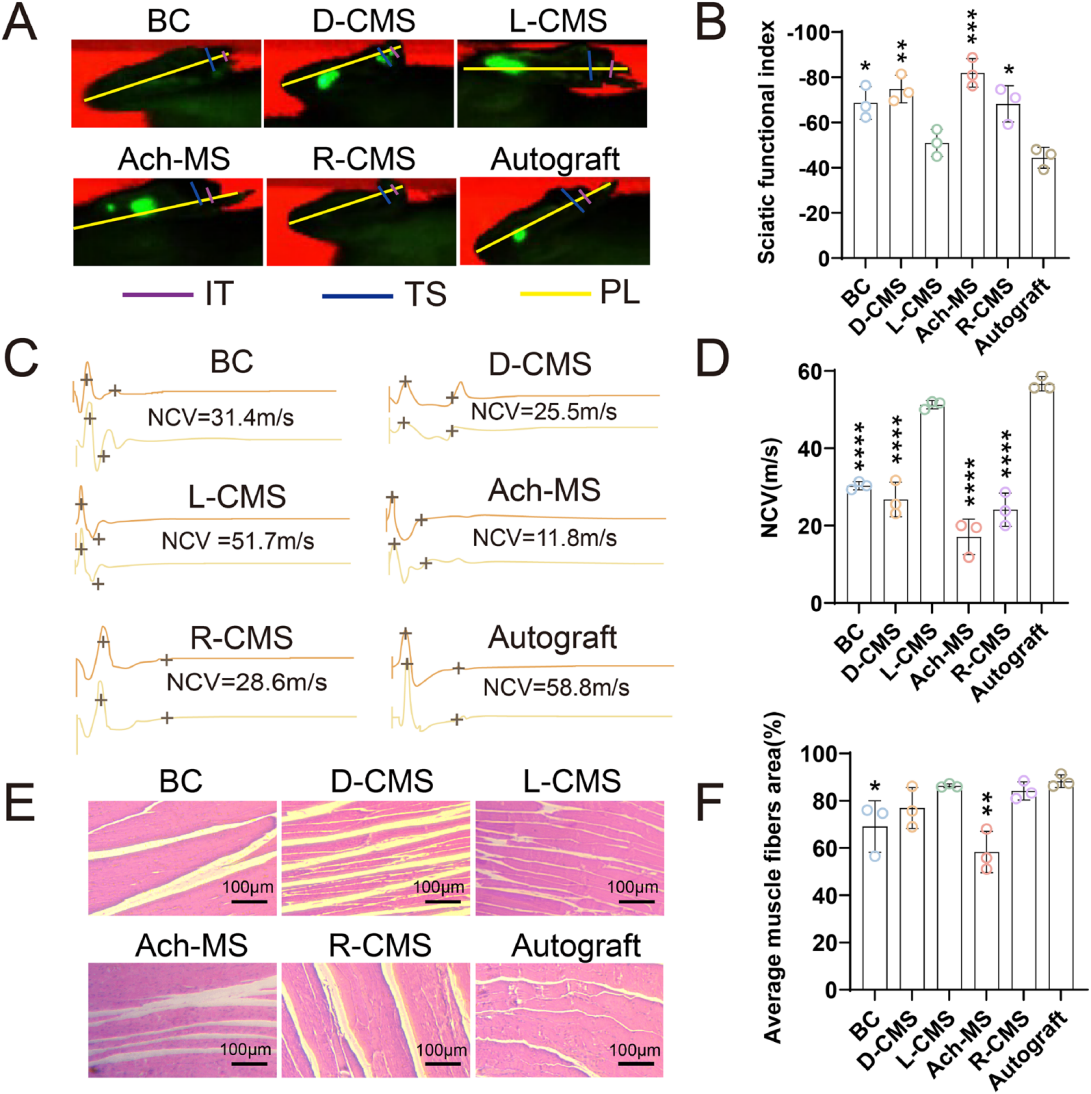
Nerve and muscle regeneration of BC, D-CMS, L-CMS, Ach-MS, R-CMS and autograft groups 12 weeks post-surgery. **A** Right hind limb footprints. **B** SFI statistics. **C** Regenerated nerve electromyography. **D** Regenerated nerve NCV. **E** HE staining of right hind-limb gastrocnemius muscle. **F** Gastrocnemius muscle fiber area analysis (*n* = 3)

The rats were placed on the walk-track analysis system, and the recovery of motor function was analyzed using the visugate system (Fig. 7A). SFI is one of the main indicators used to assess motor function recovery. The SFIs in the L-CMS group were significantly higher than those in the BC (p = 0.0226), D-CMS (p = 0.0029), Ach-MS (p = 0.0003), and R-CMS groups (p = 0.0258) (Fig. 7B). The SFIs in the L-CMS and autograft group were -51.0 and -44.4, respectively. No significant differences were observed between the L-CMS and autograft groups (p = 0.6156), indicating that L-CMS NGCs can enhance nerve function restoration to some extent.

The NCVs were 30.3 m/s, 26.8 m/s, 51.2 m/s, 17.1 m/s, 24.1 m/s, and 56.7 m/s in the BC, D-CMS, L-CMS, Ach-MS, R-CMS, and autograft groups, respectively (Fig. 7C). Notably, the NCVs of the L-CMS group and autologous groups were similar (p = 0.2186). In contrast, the NCVs of the BC, D-CMS, Ach-CM, and R-CMS groups were lower than that of the L-CMS group (p < 0.0001) (Fig. 7D), suggesting that the L-CMS NGCs played a role in promoting the electrophysiological recovery of the regenerated nerves.

The recovery of the gastrocnemius muscles in the six groups were shown in Fig. 7E. There were no significant differences in the average muscle fiber areas between the L-CMS, D-CMS, R-CMS, and autograft groups. However, there was a notable difference in the average muscle fiber area between the L-CMS group and BC group (p = 0.0403). And there was a significant difference in the average muscle fiber area between the L-CMS group and the Ach-MS group (p = 0.0015) (Fig. 7F).

### Chiral NGCs promoted nerve regeneration

**Fig. 8:**
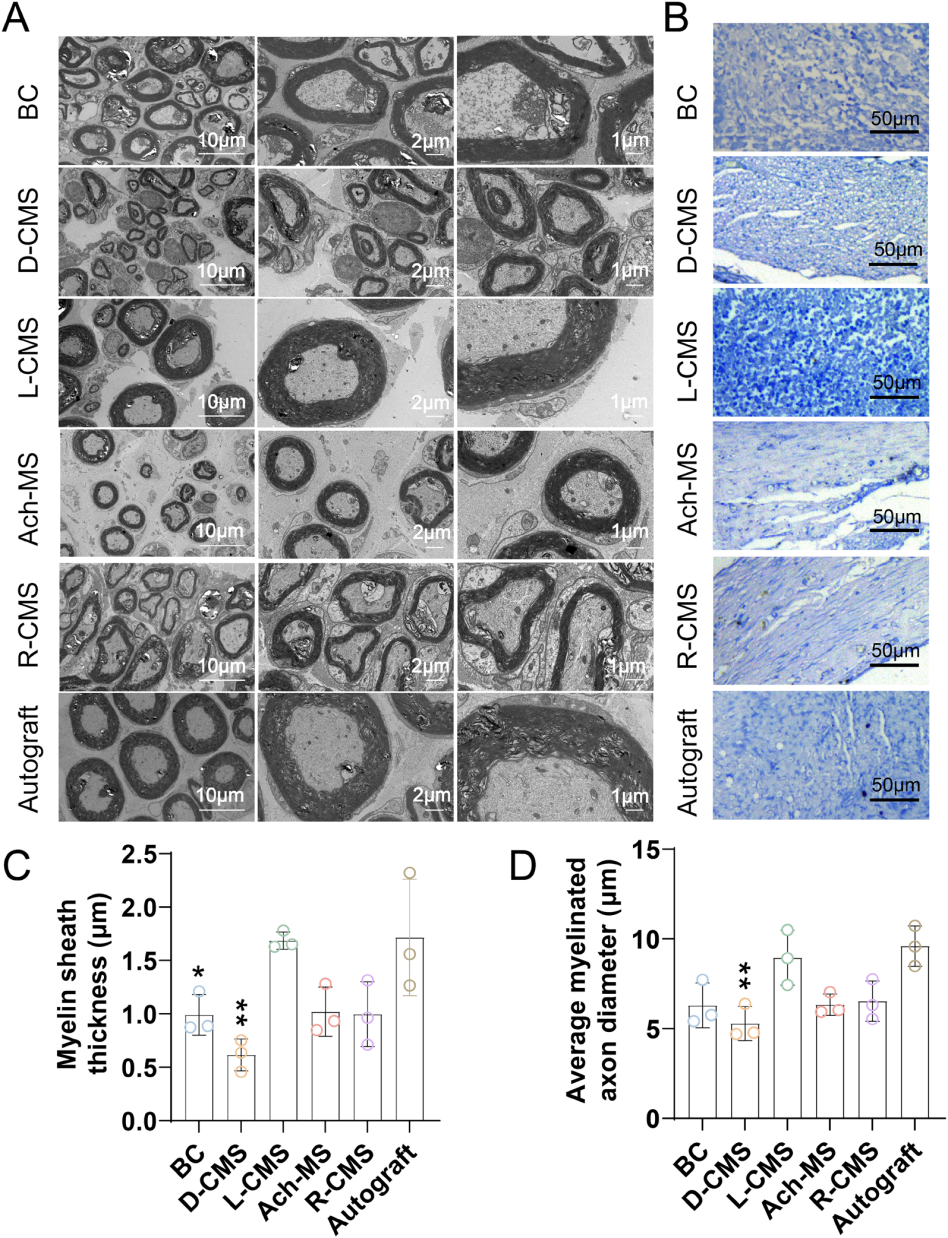
Regeneration of nerves and myelinated axons of BC, D-CMS, L-CMS, Ach-MS, R-CMS and autograft groups 12 weeks after surgery. **A** TEM of regenerated myelinated axon. **B** TB staining of regenerated nerves. **C** Average thickness of myelin sheath. **D** Average diameter of myelinated axon (*n* = 3)

After the scaffolds had been implanted in the sciatic nerve for 12 weeks, the tissue density was evaluated by TB staining of the regenerated nerves. The tissue densities of the autograft and L-CMS groups were higher than those of the other groups (Fig. 8B). The average diameter and thicknesses of the myelinated axons were also examined (Fig. 8A). Notably, the myelin sheath thicknesses was close in the L-CMS and autograft groups (p > 0.05). The myelin sheath thicknesses in the L-CMS group were not significantly different from those in the Ach-MS (p = 0.0594) and R-CMS (p = 0.0500) groups but were significantly higher than those in the BC (p = 0.0478) and D-CMS (p = 0.0030) groups (Fig. 8C). Moreover, the diameters of the myelinated axons in the L-CMS group were not significantly different from those in the BC (p = 0.0530), Ach-MS (p = 0.0570), R-CMS (p = 0.0828), and autograft (p = 0.9289) groups, whereas they were significantly better than those in the D-CMS group (p = 0.0077) (Fig. 8D).

## Discussion

We fabricated the CMS@BC scaffolds using a hydrothermal method. These scaffolds exhibit good biocompatibility and mechanical properties. Crucially, MoS_2_ exhibits good biocompatibility [48], mechanical properties [49], and electrical conductivity [24].

The therapeutic effects of MoS_2_ on tumors and neurodegenerative diseases have been reported [50–53]. However, its application in peripheral nerve regeneration has not been studied. Therefore, we evaluated the safety and efficacy of CMS@BC scaffolds for peripheral nerve regeneration. The results of the live/dead cell experiments and Annexin V-FITC apoptosis assays revealed that the toxicity of the five groups of scaffolds to SCs was negligible. In addition, the HE staining results of the main organs showed that the CMS@BC scaffolds had good biocompatibility in vivo. Collectively, these chiral scaffolds exhibit good biocompatibility in vivo and in vitro. Moreover, we performed assessments of the functional recovery, electrophysiological, morphological, myelination, and axon regeneration. The electrophysiological assay results and functional recovery were similar between the L-CMS NGCs and autograft groups. In general, the therapeutic effect of the L-CMS NGCs was better than those of the other NGCs.

Recently, transition metal dichalcogenides (TMDs) have been studied in various fields [54], and several studies have demonstrated that TMDs exhibit good biocompatibility. For example, Shah et al. showed that MoS_2_ nanosheets have low toxicity in rat pheochromocytoma cells, which forbode MoS_2_-based materials’ promising application in the field of nerve regeneration [55]. Nazari et al. demonstrated that nylon/MoS_2_ nanofibers promoted the attachment of mouse embryonic cardiac cells [56]. Ma et al. showed that a sodium alginate-MoS_2_ hydrogel significantly enhanced the proliferation of HUVECs [30]. We found that the implantation of the MoS_2_ scaffolds into rat sciatic nerve defect sites for 12 weeks did not cause illness or death, indicating that it was a relatively safe material. We also noted that the L-CMS NGCs were better than D-CMS NGCs for promoting peripheral nerve regeneration, and the reasons for this can be explained as follows. There is enantiomer selectivity between chiral nanostructures and cells [57–59]. Furthermore, there are stereoselective interactions between chiral materials and proteins. Zhao et al. demonstrated that the L-penicillamine-nanoparticle films can accelerate cell proliferation, whereas the D-penicillamine-nanoparticle films have the opposite effect [60]. Several studies have demonstrated that the left-handed materials enhanced cell adhesion and proliferation by regulating protein adsorption [61, 62]. Studies have shown that enantiomer-modified chiral surfaces can mediate cell adhesion, spreading and differentiation [63]. In particular, M2 macrophages are important for the reconstruction of the immune microenvironment during tissue injury and repair [8]. For example, Qian et al. showed that enhancing the polarization of M2 macrophages and reducing the inflammatory response are crucial for regulating the immune balance in peripheral nerve regeneration [64]. As we know, M2 polarization of the macrophage has advantages for tissue repair including nerve tissues. Xu et al. demonstrated that left- and right-handed gold biomimetic nanoparticles showed different  in vitro and in vivo immune responses, the left-handed enantiomers associate with these adhesion G-protein-coupled receptors family receptors more strongly than do the right-handed ones [67]. Furthermore, Mu et al. showed that the chiral tetrapeptide nanofibers can serve as a defense mechanism in the restoration of tissue homeostasis by upregulating macrophage M2 polarization via the Src-STAT6 axis. And the L-chirality exhibited a more potent effect in inducing macrophage M2 polarization than does the D-chirality, leading to enhanced tissue reconstruction [68], indicating that chiral materials can promote tissue repair through immunomodulation. Sun et al. showed that macrophages exhibited distinct differences in the adhesion and activation behaviors on the different enantiomers’ modified surfaces [69]. Jiang et al. demonstrated that left-handedness nanofibrils displayed higher stereo-affinity to cellular binding, macrophage M2 polarization was then promoted through a series of reactions [65]. Yang et al. demonstrated that left-handed self-assembling matrixes displayed higher stereo-affinity to cellular binding, which enhanced the clustering of mechanosensitive integrin *β*1 and activated focal adhesion kinase and Rho-associated protein kinase, as well as down-streamed PI3K/Akt1/mTOR signaling axes to promote M2 polarization [66]. Overall, we believe that L-CMS NGCs promote peripheral nerve regeneration by promoting M2 macrophage polarization.

Moreover, angiogenesis can promote the repair of injured peripheral nerves [70]. Cattin et al. showed that blood vessels were necessary and sufficient to guide the migration of SCs during the peripheral nerve regeneration [71]. Liu et al. showed that left-handed helical nanofibers promoted HUVECs adhesion and proliferation [72]. Xing et al. constructed chiral HA-LM2-RMR fibers that can promote angiogenesis by upregulating VEGF and OPA1 expression [73]. We believe that another reason for the greater effectiveness of L-CMS NGCs in promoting nerve regeneration is their capacity to facilitate angiogenesis.

## Conclusion

In summary, we fabricated CMS@BC scaffolds using a hydrothermal approach.  These chiral scaffolds exhibited good mechanical stability and hydrophilicity. The results of *vitro* experiments showed that the L-CMS scaffolds were better than the other scaffolds in promoting SCs proliferation. After the scaffolds had been implanted in rat sciatic nerves for 12 weeks, it was found that the scaffolds have enhanced the axonal myelination and stimulated the electroactivity of the regenerated nerves. Overall, the L-CMS scaffolds contributed to functional restoration and nerve regeneration. Our study provides a significant reference for chiral nanomaterials in peripheral nerve regeneration.

### Supplementary Information

**Additional file 1: Fig. S1.** Mechanical properties of BC, D-CMS, L-CMS, Ach-MS and R-CMS materials. **A** Tensile strength of BC, D-CMS, L-CMS, Ach-MS and R-CMS materials. **B** Stress-strain curves for BC, D-CMS, L-CMS, Ach-MS and R-CMS materials. **C** Stress profiles of BC, D-CMS, L-CMS, Ach-MS and R-CMS materials with time. **Fig. S2.** Immunofluorescence of SCs on BC, D-CMS, L-CMS, Ach-MS and R-CMS scaffolds. **A** CJun immunofluorescence. **B** Relative levels of CJun (*n* = 3). **Table S1.** Experimental reagents. **Table S2.** Experimental Instruments. **Table S3.** Mechanisms of different types of nerve conduits promoting peripheral nerve regeneration.

## Data Availability

All data that support the findings of this study are included in the paper and the Supporting Information. Additional data related to this paper are available from the corresponding author upon reasonable request.

## Abbreviations

MoS_2_: Molybdenum disulfide
CMS: Chiral MoS_2_
BC: Bacterial cellulose
SCs: Schwann cells
NGC: Nerve guidance conduit
NSC: Neural stem cell
HUVECs: Human umbilical vein endothelial cells
AuNP: Gold nanoparticle
SCI: Spinal cord injury
PFA: Paraformaldehyde
SFI: Sciatic functional index
NCV: Nerve conducting velocity
TEM: Transmission electron microscopy
HE: Hematoxylin and eosin
ANOVA: Analysis of variance
APP: 2-Amino-3-phenyl-1-propanol
SEM: Scanning electron microscopy
