# Supplementary material for: Chiral MoS2@BC fibrous membranes selectively promote peripheral nerve regeneration
Source: J Nanobiotechnology. 2024 Jun 17;22:337. doi: 10.1186/s12951-024-02493-6 (PMC11181549; doi:10.1186/s12951-024-02493-6)
Supplement: Supplementary file 1 — Additional file 1: Fig. S1. Mechanical properties of BC, D-CMS, L-CMS, Ach-MS and R-CMS materials. A Tensile strength of BC, D-CMS, L-CMS, Ach-MS and R-CMS materials. B Stress-strain curves for BC, D-CMS, L-CMS, Ach-MS and R-CMS materials. C Stress profiles of BC, D-CMS, L-CMS, Ach-MS and R-CMS materials with time. Fig. S2. Immunofluorescence of SCs on BC, D-CMS, L-CMS, Ach-MS and R-CMS scaffolds. A CJun immunofluorescence. B Relative levels of CJun (n = 3). Table S1. Experimental reagents. Table S2. Experimental Instruments. Table S3. Mechanisms of different types of nerve conduits promoting peripheral nerve regeneration. [file 12951_2024_2493_MOESM1_ESM.docx]

**Supplementary material**


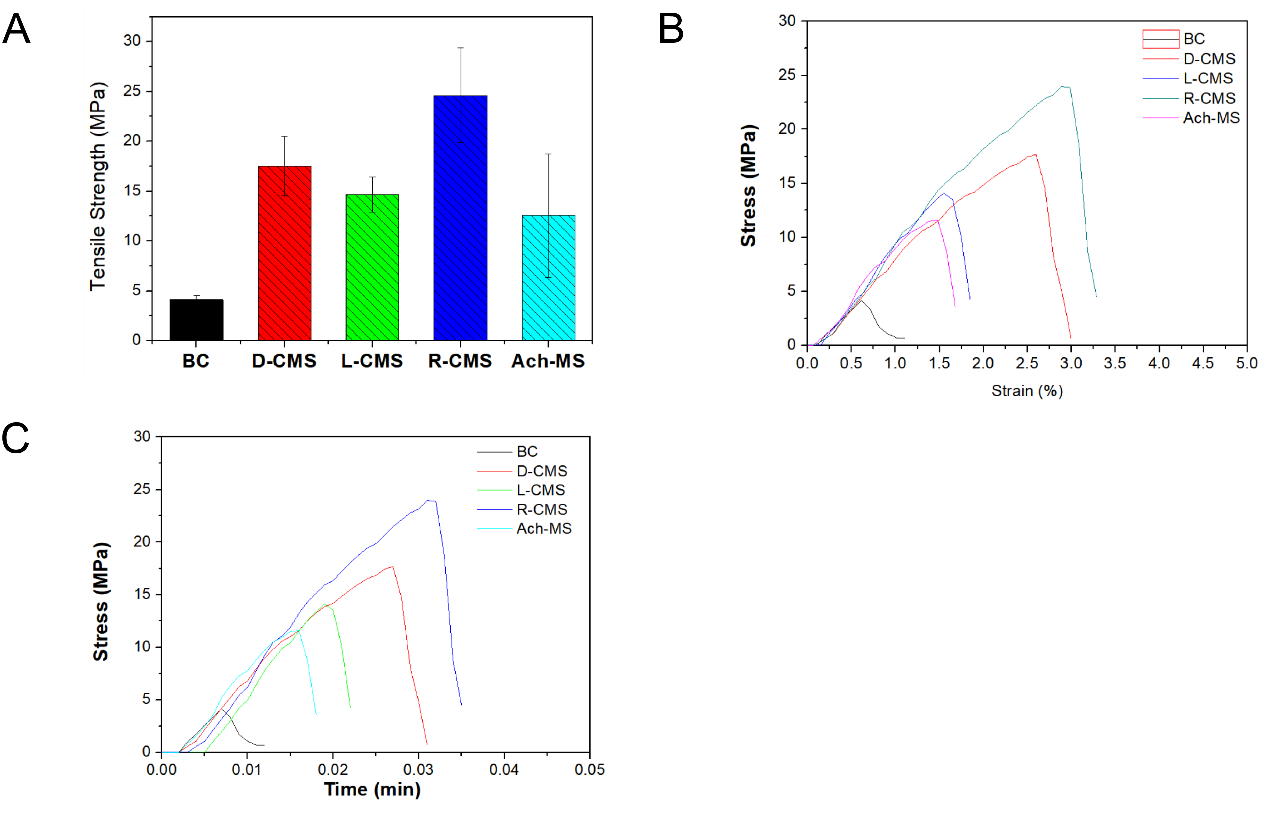


**Fig. S1** Mechanical properties of BC, D-CMS, L-CMS, Ach-MS and R-CMS materials. (A) Tensile strength of BC, D-CMS, L-CMS, Ach-MS and R-CMS materials. (B) Stress-strain curves for BC, D-CMS, L-CMS, Ach-MS and R-CMS materials. (C) Stress profiles of BC, D-CMS, L-CMS, Ach-MS and R-CMS materials with time.


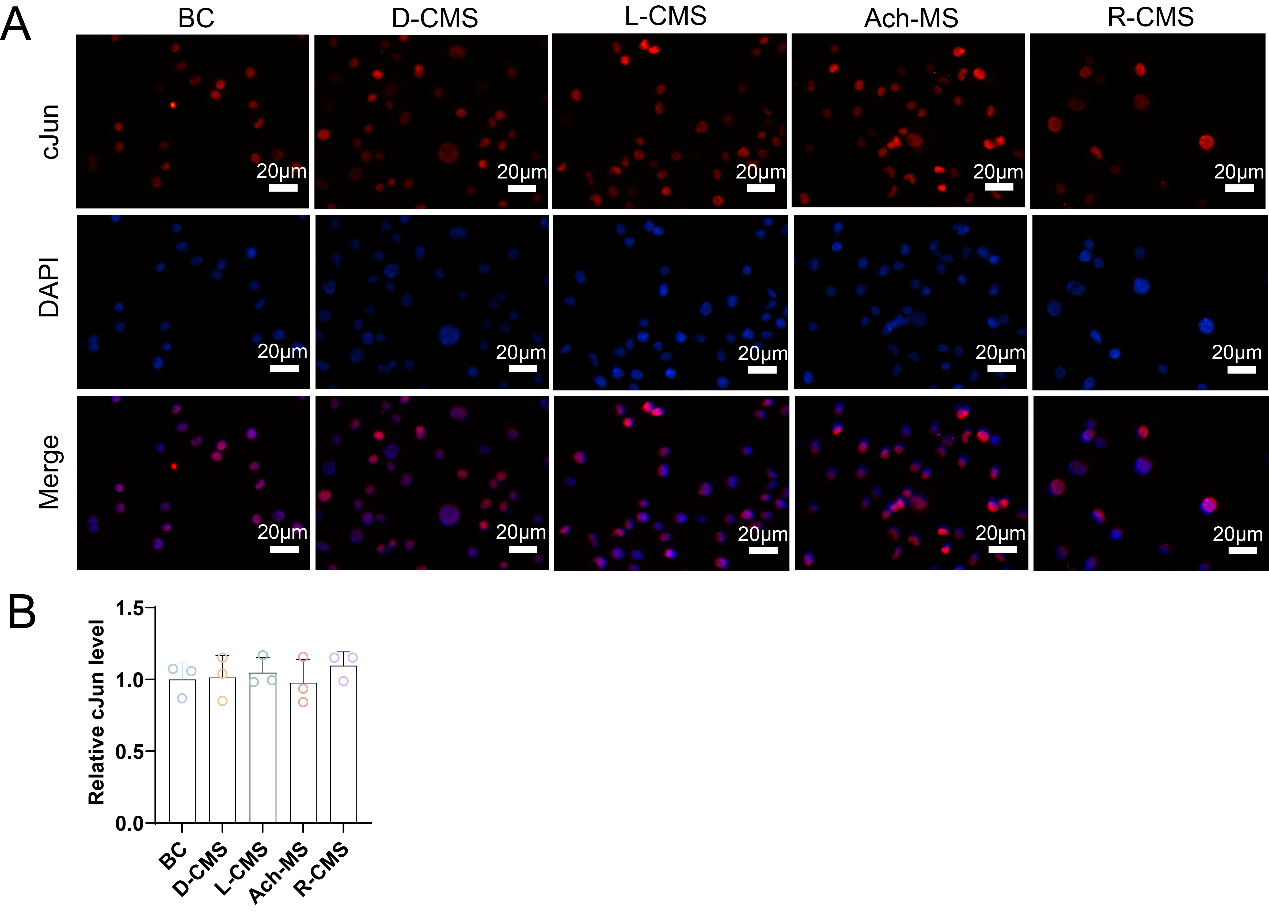


**Fig. S2** Immunofluorescence of SCs on BC, D-CMS, L-CMS, Ach-MS and R-CMS scaffolds. (A) CJun immunofluorescence. (B) Relative levels of CJun (*n* = 3).

All the codes of the products we used and the specific models of the instruments are shown below.

**Supplementary Table 1** Experimental reagents.

| Experiment reagent | Code |
| --- | --- |
| Thioacetamide (CH_3_CSNH_2_; 99.0%) | Merck (T818853) |
| Sodium molybdate dihydrate (Na_2_MO_3_·2H_2_O, 99.0%) | Merck (S817613) |
| D-2-amino-3-phenyl-1-propanol (99.9%) | Tansoole (01083250) |
| L-2-amino-3-phenyl-1-propanol (99.9%) | Tansoole (01060193) |
| Racemic-2-amino-3-phenyl-1-propanol (99.9%) | Tansoole (01083250)/ Tansoole (01060193) |
| BC fibrous membranes | Guilin Qihong (QH256418-32) |
| RSC 96 cells | Shanghai Zhongqiaoxinzhou Biotech (ZQ 0154) |
| Dulbecco’s Modified Eagle Medium | Gibco (C11995500BT) |
| Fetal bovine serum | Gibco (10099158) |
| penicillin/streptomycin solution | Gibco (15140122) |
| 0.25% Trypsin-EDTA | Gibco (25200-056) |
| PBS | Servicebio (G4202) |
| Cell Counting Kit-8 | NCM Biotech (C6005)/Absin (abs50003) |
| Calcein-AM/PI Kit | Solarbio (CA1630) |
| Annexin V-FITC apoptosis assay kit | Absin (abs50001) |
| Anti-Ki67 antibody | Abcam (ab16667) |
| Anti-c-Jun antibody | Abcam (ab40766) |
| Anti-S100 beta antibody | Abcam(ab52642)/proteintech(15146-1-AP) |
| Cy3-Goat anti-Rabbit IgG | Simuwubio (SD0138) |
| Albumin Bovine V | Solarbio (A8020) |
| Triton X-100 | Solarbio (T8200) |
| 4% paraformaldehyde | Servicebio (G1101)/Biosharp (23286549) |
| FITC Phalloidin | Yeasen (40735ES75) |
| DAPI Staining Solution | Beyotime (C1005) |

The instruments used in the experiment are shown in the following table:

**Supplementary Table 2** Experimental Instruments.

| Instruments | Code |
| --- | --- |
| Scanning electron microscope | JEOL (JSM-7900F) |
| Contact angle measurement | SINDIN (SDC-350H) |
| Hydrothermal synthesis reactor | Hefei Kejing (50ml) |
| Circular dichroism spectrometer | JASCO (J-1500) |
| X-ray Powder diffractometer | Bruker (D2) |
| Tensile testing machine | Tophung (TH-8100ST) |
| Phase contrast microscope | Leica (DMIL LED) |
| Fluorescence microscope | Leica (DMi8) |
| High speed centrifuge | Beckman Coulter (20140177) |
| -80 ℃ medical cryogenic box | Thermo (FDE40086FV) |
| Flow cytometer | BD FACSCelesta (660344) |

**Supplementary Table 3** Mechanisms of different types of nerve conduits promoting peripheral nerve regeneration.

| Serial num-ber | Scaffold | Therapeutic outcome | Mechanism |
| --- | --- | --- | --- |
| 1 | Bio three-dimensional conduit | The regenerated nerve in the Bio 3D group was significantly superior to that in the silicone group based on morphology, kinematics, electrophysiology, and wet muscle weight. | By inducing angiogenesis. |
| 2 | Black phosphorus /PCL | The black phosphorus nano scaffold induced angiogenesis and neurogenesis and stimulated calcium-dependent axon regrowth and remyelination. | By inducing angiogenesis. |
| 3 | longitudinally oriented collagen hydrogel-grafted elastic nerve guidance conduits | The scaffolds could promote axonal regeneration and remyelination, and promote good functional recovery. | The aligned collagen hydrogels provide a preferable environment for nerve regeneration, functioning as an oriented guidance path. |
| 4 | anisotropic micro-nanocomposite | The scaffolds could effectively induce the orientation growth of Schwann cells and up-regulate the genes and proteins relevant to myelination. | Promoting nerve regeneration by simulating the regenerative microenvironment. |
| 5 | Nerve conduits composed of high-resolution anisotropic microfiber grid-cordes with randomly organized nanofiber sheaths | The newly regenerated nerve tissue that formed within the composite nerve conduits showed restored neurological functions that were superior compared to sheaths-only scaffolds and Neurolac nerve conduit controls. | Using synthetic biophysical cues to promote nerve regeneration. |
| 6 | Human umbilical cord mesenchymal stem cells -ECM modified PCL-SF electrospinning materials | The dual-bionic material exerted a similar effect to that of autologous nerve transplantation in bridging peripheral nerve defects in rats. | Promoting nerve regeneration by mimicking the peripheral neural microenvironment. |
| 7 | Dimethyl fumarate/ regenerated silk fibroin/ poly(3,4-ethylenedioxythiophene): poly (styrene sulfonate) conduit | Promoting the functional recovery of neurons. | By inhibiting SCs pyroptosis and reducing inflammatory factor release, shifting macrophage polarization from the inflammatory M1 phenotype to the tissue regenerative M2 phenotype. |
| 8 | 3D melatonin/polycaprolactone nerve guide conduit | 3D manufacture of melatonin/polycaprolactone nerve guide conduit increased Schwann cell proliferation and neural expression in vitro and promoted functional, electrophysiological and morphological nerve regeneration in vivo. | Inhibiting oxidative stress and inflammation after traumatic insults. |
| 9 | Bionic peptide hydrogel scaffold | The bionic scaffold promoted M2 transformation in situ and led to proliferation and migration of Schwann cells, neuron growth and motor function recovery. | By remodeling the local environment for M2 transformation and recruitment. |
| 10 | Poly(ɛ-caprolactone)/carbon nanotubes composite fiber | Promoting the oriented growth of neural cells in vitro as well as the regeneration of injured sciatic nerves in vivo. | By aligned topography and electrical stimulation to promote neural regeneration. |
| 11 | Poly-pyrrole/silk fibroin conductive composite scaffold | Promoting SCs viability, proliferation and migration, as well as upregulated expression of neurotrophic factors. Promoting axonal regeneration and remyelination in vivo. | Promoting peripheral nerve regeneration by electrical stimulation. |
| 12 | R (graphene oxide / gelatin- methacrylate) | Improving muscle weight increase, electro-conduction velocity, and sciatic nerve function index. | Promoting peripheral nerve regeneration by delivering electrical signals. |
